# The Psychological Impact of Receiving a Biomarker Informed ADRD Diagnosis in a Real‐World Sample: the PARADE Study

**DOI:** 10.1002/alz70858_105907

**Published:** 2025-12-26

**Authors:** Melissa L Knox, Megan G Witbracht, Josh D Grill, Jennifer H Lingler

**Affiliations:** ^1^ University of Pittsburgh Alzheimer's Disease Research Center (ADRC), Pittsburgh, PA, USA; ^2^ Institute for Memory Impairments and Neurological Disorders, University of California, Irvine, Irvine, CA, USA; ^3^ University of California, Irvine, Irvine, CA, USA; ^4^ The UC Irvine Institute for Memory Impairments and Neurological Disorders, Irvine, CA, USA

## Abstract

**Background:**

The increasing use of biomarkers for Alzheimer's disease has improved diagnostic outcomes but the psychological and behavioral impact of these biomarker informed diagnoses on families seeking care is not well studied. We developed the Patient And family member Reactions to biomarker informed ADRD DiagnosEs (PARADE) Study to better understand these reactions in a real‐world population.

**Method:**

Participants from New IDEAS, a coverage with evidence development study of amyloid PET, were invited to enroll in PARADE, an ongoing 6‐month longitudinal study designed to measure the responses to receiving an ADRD diagnosis in people living across the US. We conducted interviews remotely using telephone or video conferencing at 4 time points from pre disclosure of results to 24‐weeks post‐disclosure. Intrusive thoughts were measured with the Impact of Events Scale (IES). Controlling for age, sex, and race, we assessed differences in IES by amyloid status at the 4‐week post disclosure visit, both as a continuous outcome using bootstrap linear regression and as a categorical outcome using logistic regression, with the latter based on the clinical threshold of IES>26.

**Result:**

We describe findings from a preliminary analysis of participants (*n* = 52) referred from New IDEAS. Most were female (56.9%), married (68.6%), retired (86.3%), and had at least 12 years of education (84.3%). Fourteen people self‐reported Hispanic ethnicity; 33 identified as White race and 14 identified as Black race, while 5 people reported another race or did not report race. Participants were distributed across quintiles of area deprivation index, although the sample skewed towards lower deprivation (1=57.1%, 2=22.5%, 3=6.1%, 4=2.0%, 5=12.2%). The adjusted mean IES score was 22.54 ± 2.71 for the amyloid‐positive group (*n* = 30) and 14.76 ± 3.11 for the amyloid‐negative group (*n* = 22). This difference trended toward significance (*p* = 0.058). We did not observe a difference in the odds of experiencing clinically significant intrusive thoughts between the groups.

**Conclusion:**

These data suggest that in the weeks after disclosure, patients who receive a positive amyloid PET result that supports an Alzheimer's diagnosis may experience greater subclinical distress compared to their not elevated counterparts.

